# Supplementary material for: Optical Neuroimage Studio (OptiNiSt): Intuitive, scalable, extendable framework for optical neuroimage data analysis
Source: PLoS Comput Biol. 2025 May 19;21(5):e1013087. doi: 10.1371/journal.pcbi.1013087 (PMC12124740; doi:10.1371/journal.pcbi.1013087)
Supplement: S1 Table — (PDF) [file pcbi.1013087.s001.pdf]

Input modules (specify input files)

image: input as movie images (multi-page, multi-channel .tiff files are supported)
csv: 1or 2 dimensional table (with or without column names)
hdf5: hierarchical binary data inlcuding .nwb
behavior: 1or 2 dimensional table (with or without column names) as a behavioral parameters
fluo: csv file as a fluorescence data
matlab: an array or a matrix
microscope: input image files in Inscopix (.isxd), NIKN (.nd2), or Olympus (.oir) format.

Upstream modules (perform cell extraction and spike deconvolution)

CaImAn
caiman_mc: motion correction
caiman_cnmf: cNMF (cell extraction)
caiman_cnmfe: cNMFE(cell extraction for endoscope data)
caiman_multisession: cNMF or cNMFE for multisession data

Suite2p
suite2p_file_convert: initialization
suite2p_registration: motion correction
suite2p_roi: cell extraction
suite2p_spike_deconv: spike deconvolution

LCCD
lccd_cell_detection: cell extraction

Downstream modules (analysis of population activities)

basic neural analyses
eta: event-triggered averaging of individual neuronal activity
correlation**: pairwise correlation across inputs**
cross_correlation: cross correlation across inputs

dimension reduction
pca: principal component analysis
dpca: demixed-principal component analysis
cca: canonical correlation analysis
tsne: t-distributed stochastic neighbor embedding

causality analyses
granger: granger causality analysis across inputs

decoding/encoding
svm: support vector machine
lda: linear discriminant analysis
glm: generalized linear model

Utility modules

microscope_to_img: Conversion of the microscope module output to image format
roi_from_hdf5: Extraction of ROI information from hdf5 (nwb) input and visualization
fluo_from_hdf5: Extraction of fluorescence from hdf5 (nwb) input and visualization
roi_fluo_from_hdf5: Extraction of ROI and fluorescence from hdf (nwb) input and visualization
vacant_roi: Manual selection of ROI areas and calculation of mean fluorescence within them
