# Supplementary material for: Optical Neuroimage Studio (OptiNiSt): Intuitive, scalable, extendable framework for optical neuroimage data analysis
Source: PLoS Comput Biol. 2025 May 19;21(5):e1013087. doi: 10.1371/journal.pcbi.1013087 (PMC12124740; doi:10.1371/journal.pcbi.1013087)
Supplement: S1 Text — (DOCX) [file pcbi.1013087.s002.docx]

**Installation (general procedure)**

Docker image and pip installation methods are provided in addition to Open codes on GitHub. Installation for Windows, Mac, and Linux is supported. We have provided two different setting modes: standalone and multiuser. The standalone mode assumes that OptiNiSt is installed on a PC and used by a single user. The multiuser mode allows authentication and use by multiple users and running of multiple workflows simultaneously, providing a single platform for using a high performance server machine for individual or collaborative analyses. OptiNiSt uses mysql or mariadb for the management of accounts, and Firebase Authentication, for authentication. Installation of both modes is simple, and the procedure is documented (<https://optinist.readthedocs.io/en/latest/>).

**Dataset for the examples**

We used 2-photon imaging data obtained from the parietal area of a mouse during auditory stimulation from 12 speakers surrounding the animal. We prepared cropped small size imaging data (128 pixels x 128 pixels x 3000 frames, 100 MB) and behavior data for the demonstration, and the large size original data (512 x 512 x 22500, 12 GB) were used for confirming that OptiNiSt can handle an original-size dataset. Both datasets are available at Zenodo repository.

Zenodo repository: https://doi.org/10.5281/zenodo.13357960.
