# Supplementary material for: Optical Neuroimage Studio (OptiNiSt): Intuitive, scalable, extendable framework for optical neuroimage data analysis
Source: PLoS Comput Biol. 2025 May 19;21(5):e1013087. doi: 10.1371/journal.pcbi.1013087 (PMC12124740; doi:10.1371/journal.pcbi.1013087)
Supplement: Attachment — Submitted filename: optinist review.pdf [file pcbi.1013087.s011.pdf]

I have divided my review into 3 sections, general comments, specific areas for improvement, and my attempt at using the software.

General comments and questions

This is really a server-side application, please take a look at NeuroCAAS which seems similar and compare optimist with NeuroCAAS:
https://www.sciencedirect.com/science/article/pii/S0896627322005876

Does optinist use lazy loading, how does it handle large movies?

Use of NWB is good for interoperability.

The introduction is often quite repetitive and can be made much more concise.

The paper's comments on Mesmerize:

Mesmerize [15] is written fully in Python, making it readily available, but the desktop graphical user interface (GUI) is not supported any more.

As the main developer of mesmerize the reasons for this are the following, and important to keep in mind for anyone developing end-user tools in the calcium imaging community:

- Long-term maintenance of a GUI tool that works cross-platform is very challenging, especially considering the sub-optimal maintenance of the domain-specific packages that you rely on and sometimes the excessive dependencies that these packages have.
- From our experience providing a GUI in the form of a flowchart for downstream analysis in the old mesmerize mostly led users to creating things that *looked* like they made sense without really understanding the underlying analysis. From our experience through teaching scientists at various institutions, we found that the barrier for users was on the side of understanding downstream analysis rather than basic programming.
- From our experience, the most useful component of mesmerize is performing a parameter search for caiman NoRMCorr and CNMF(E). The scope for downstream is too broad and has to be tailor-made to each unique biological question. This is why we now strongly encourage users towards other tools, such as pynapple, for downstream analysis.
- You risk scope explosion by trying to do everything. Given the challenging space of the calcium imaging analysis ecosystem, if you are building upon existing domain-specific software it is usually more practical to create a tool that does only one or a few things very well.

Areas for improvement

- What is the long term maintenance plan? It is often the case that scientific software is published, and then the authors move on and the software is abandoned.

- There are pinned dependencies and more worrisome is the pin to python 3.8, example: https://github.com/oist/optinist/blob/main/studio/app/optinist/wrappers/caiman/conda/caiman.yaml
    - This is a critical issue for long-term maintenance.
    - Python 3.8 is past the EOL: https://devguide.python.org/versions/
- Is there a way to perform a parameter grid search without clicking and adding each parameter variant manually?
- Caiman has nearly 100 parameters, while some of the most common ones are shown in the UI. In order to be useful for a diverse range of datasets it is necessary to be able to supply all the other params. Perhaps have a large text entry area where users can input additional params in the form of a dict or yaml?
- I noticed you have a "use_cuda" option for caiman NoRMCorre. I would suggest removing it to avoid user confusion since caiman's GPU accelerated NoRMCorre was written using skcuda which is no longer maintained.

Use of optinist

Docker installation was seamless following these instructions:
https://optinist.readthedocs.io/en/latest/installation/docker.html
I have Docker version 24.0.2 which was released in May 2023, so relatively recent.

I tried to use it, I gave it the caiman demo movie and tried to do motion correction, this was the workflow:

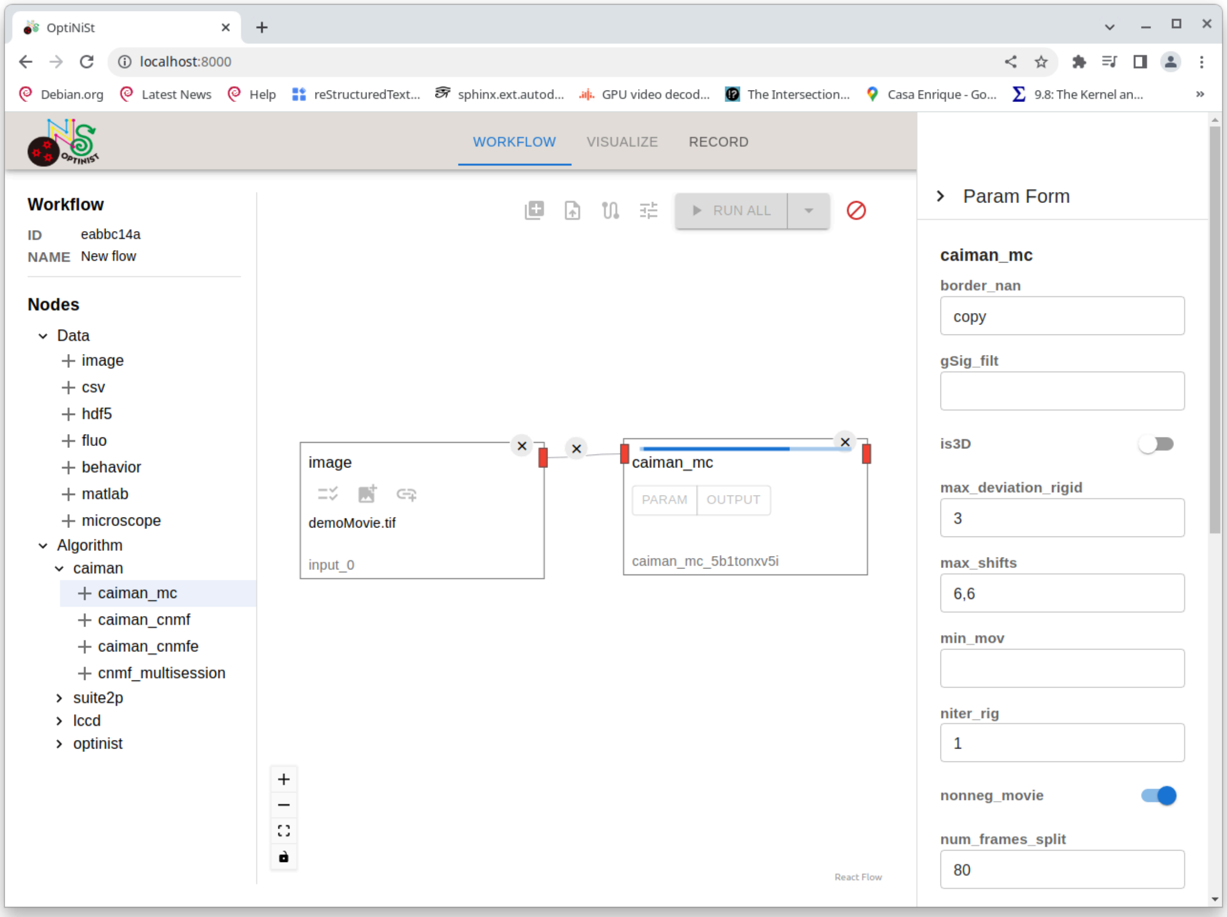

The caiman demo movie I'm referring to:
https://github.com/flatironinstitute/CaImAn/blob/main/example_movies/demoMovie.tif

I clicked "Run all" to run the workflow, but unfortunately I could not get further. I first thought it hanged for some reason because even after 10 minutes it didn't finish. This movie usually finishes within seconds on my computer. ~15 minutes later it actually didn't finish and this is the traceback I got:

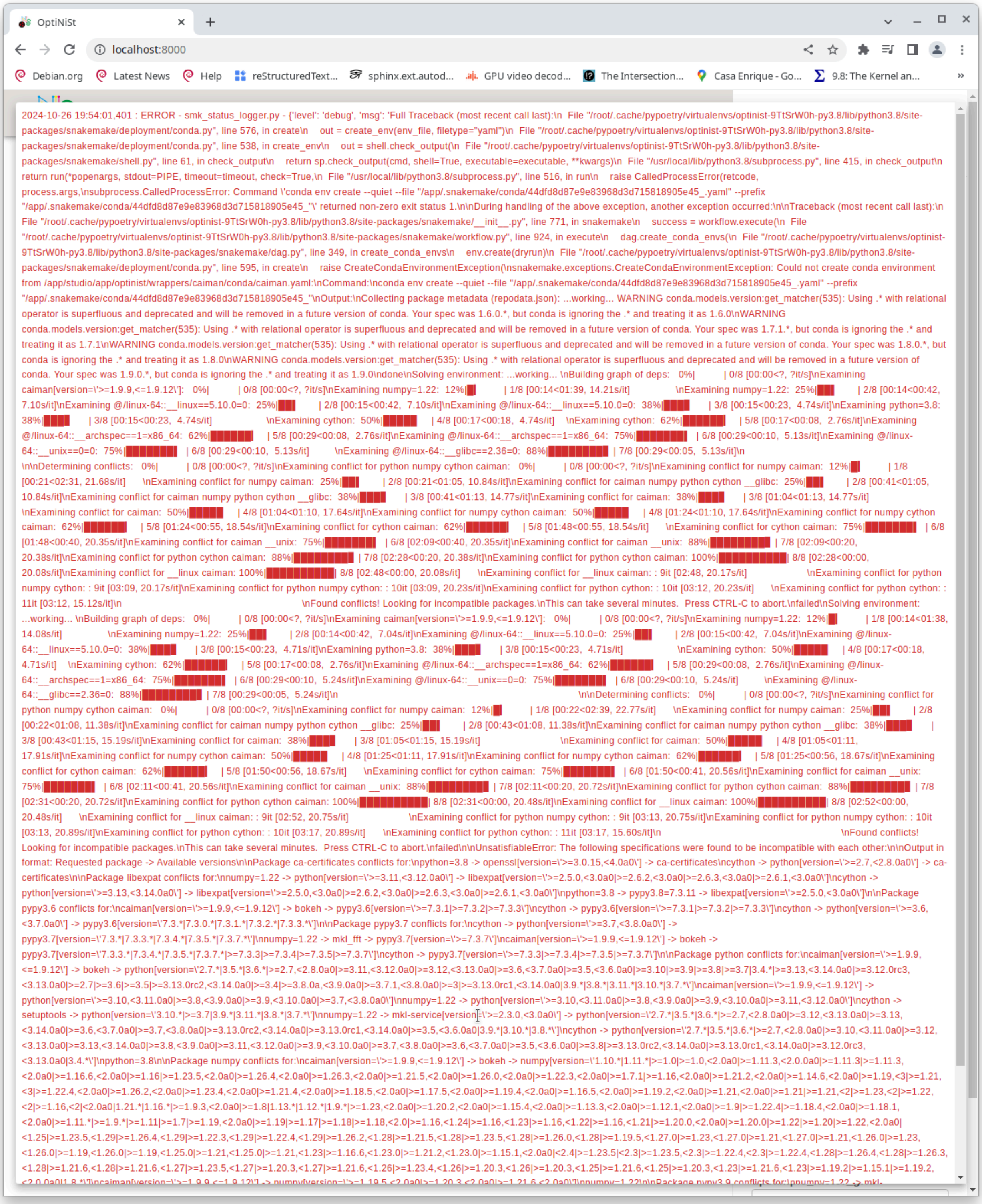

I have important suggestions to improve this experience:

1. Have a way to install the components, such as caiman, *before* running the workflow so that the user knows if the install will work. I thought that after pulling the docker image I was ready to go.
2. Format the traceback display and the output to `error.log` so that they are human readable. Everything is just output as one line which makes it very difficult to read.
3. I've attached the full `error.log` with this review.
4. Have a way for users to easily monitor the output while a workflow is running.

About the error itself, we can see that conda failed to create an environment. This is probably due to the pinned dependencies mentioned earlier, and being pinned to python3.8. I didn't go through it fully but there seem to be a lot of dependency conflicts.

Unfortunately I couldn't not try the software further due to these issues. If the developers can address these issues I am happy to make another attempt to properly evaluate optimist from a user's perspective.

With that said, I do still support publication of this manuscript after these issues have been addressed. Maintenance of scientific software is hard, and it is especially difficult in the calcium imaging community.

Other comments:

I couldn't find any way to set the number of processes to use when running a workflow, this is quite essential.

I wouldn't put more effort into making it possible for users to add their custom modules. From our experience users who are comfortable with programming are more likely to just write their own scripts or notebooks than trying to do that within the constraints of any existing toolkit. This also ties into creating tools with well-defined scope rather than a tool that tries to do everyting.
