# Supplementary material for: Optical Neuroimage Studio (OptiNiSt): Intuitive, scalable, extendable framework for optical neuroimage data analysis
Source: PLoS Comput Biol. 2025 May 19;21(5):e1013087. doi: 10.1371/journal.pcbi.1013087 (PMC12124740; doi:10.1371/journal.pcbi.1013087)
Supplement: Attachment — Submitted filename: Response to Reviewers.docx [file pcbi.1013087.s008.docx]

PCOMPBIOL-D-24-01493

Optical Neuroimage Studio (OptiNiSt): intuitive, scalable, extendable framework for optical neuroimage data analysis

PLOS Computational Biology

**Response to the reviewer 1**

I have divided my review into 3 sections, general comments, specific areas for improvement,

and my attempt at using the software.

Thank you very much for your insightful comments and suggestions. We understand that Mesmerize had already been under development when we started working on OptiNiSt, and it was developed with a very similar concept. Your comments, based on your development experience, are highly persuasive and valuable.

General comments and questions

1. This is really a server-side application, please take a look at NeuroCAAS which seems similar

and compare optimist with NeuroCAAS:

https://www.sciencedirect.com/science/article/pii/S0896627322005876

We added the description of NeuroCAAS and clarified the difference between NeuroCAAS and OptiNiSt (line 90 (Introduction) and line 529 (future direction)).

2. Does optinist use lazy loading, how does it handle large movies?

Regarding data visualization and plotting, we have implemented lazy loading. For example, when visualizing a movie for checking purposes, only the first 10 frames are loaded by default. Similarly, for displaying extracted time courses, only the fixed number of time points are shown by default. Both cases are designed to allow reloading of specific portions of the data as needed.

3. Use of NWB is good for interoperability.

We believe so.

4. The introduction is often quite repetitive and can be made much more concise.

We have rewritten the Introduction to make it more concise.

The paper’s comments on Mesmerize:

Mesmerize [15] is written fully in Python, making it readily available, but the desktop

graphical user interface (GUI) is not supported any more.

As the main developer of mesmerize the reasons for this are the following, and important to

keep in mind for anyone developing end-user tools in the calcium imaging community:

● Long-term maintenance of a GUI tool that works cross-platform is very challenging,

especially considering the sub-optimal maintenance of the domain-specific packages

that you rely on and sometimes the excessive dependencies that these packages have.

Thank you for sharing your valuable experiences with Mesmerize. In OptiNiSt, GUI frontend and computing backend are clearly separated, which makes the GUI part less dependent on the specifics of computing packages.

● From our experience providing a GUI in the form of a flowchart for downstream analysis in

the old mesmerize mostly led users to creating things that looked like they made sense

without really understanding the underlying analysis. From our experience through teaching

scientists at various institutions, we found that the barrier for users was on the side of

understanding downstream analysis rather than basic programming.

We believe that making the analysis tools easily accessible by a GUI to allow visualization of the result at each step and exploration of parameters promotes intuitive understanding of the methods. We are also planning to enhance the documentation of analysis tools and to make the description/explanation of the modules easily accessible from the GUI in the next update.

● From our experience, the most useful component of mesmerize is performing a

parameter search for caiman NoRMCorr and CNMF(E). The scope for downstream is

too broad and has to be tailor-made to each unique biological question. This is why we

now strongly encourage users towards other tools, such as pynapple, for downstream

analysis.

Thank you for pointing us to pynapple. We aim to provide easy interfaces to a selection of such tools for interactive testing.

● You risk scope explosion by trying to do everything. Given the challenging space of the

calcium imaging analysis ecosystem, if you are building upon existing domain-specific

software it is usually more practical to create a tool that does only one or a few things

very well.

Regarding the risk of scope explosion, we do not aim to include everything by ourselves. Instead, we aim to provide simple wrappers for inclusion of desired analysis modules. We also plan to promote algorithm developers to provide their methods as modules for OptiNiSt, which may facilitate their usage. In fact, several experts in the field have shown interest. In the future, we aim to increase the options of analysis modules through events like hackathons inviting these experts.

Areas for improvement

5 ● What is the long term maintenance plan? It is often the case that scientific software is

published, and then the authors move on and the software is abandoned.

OptiNiSt is supported by Japan’s Brain/MINDS 2.0 program, supported by AMED through 2030. We are going to release the next update by the end of March 2025, and aim to continue maintaining and improving the software.

While designs, specifications and testing of OptiNiSt are done by neuroscience researchers, actual coding is done by professional software development team that can create maintainable codes robust against personnel turnover, which actually happened in the course of development. We promote ease of maintenance by recording the development history. This setup is more resilient to the scenario envisioned by reviewers, where key developers are researchers or students, who would move on for their careers.

6 ● There are pinned dependencies and more worrisome is the pin to python 3.8, example:

https://github.com/oist/optinist/blob/main/studio/app/optinist/wrappers/caiman/conda/cai

man.yaml

○ This is a critical issue for long-term maintenance.

○ Python 3.8 is past the EOL: https://devguide.python.org/versions/

Even though the present OptiNiSt uses python 3.8 for CaImAn environment, which is required for CaImAn v1.9, in the next update, we plan to update CaImAn, and at that time, we will specify higher version of Python.

Snakemake allows each module to setup its own conda environment with specific versions of python and packages. It is possible to use older versions of python and packages only for the modules that require them.

7 ● Is there a way to perform a parameter grid search without clicking and adding each

parameter variant manually?

The current version of OptiNiSt does not provide a built-in method for parameter grid search, but we are aware of this limitation. At present, users can manually explore a range of parameters, save the pipeline’s Snakefile, and then create variants. Snakemake can by itself manage parallel execution of multiple threads. Additionally, we are considering introducing a feature to allow users to repeat similar processes with modified parameters via the GUI. This has been documented in the text (Limitations section line:520-).

8 ● Caiman has nearly 100 parameters, while some of the most common ones are shown in

the UI. In order to be useful for a diverse range of datasets it is necessary to be able to

supply all the other params. Perhaps have a large text entry area where users can input

additional params in the form of a dict or yaml?

In the background, all parameters are accepted as inputs; however, contrary to the reviewer's comment, there has been feedback suggesting that beginners might find the number of parameters overwhelming and difficult to understand. Therefore, the default display is limited to parameters that are relatively commonly modified. To display and make all parameters adjustable in the GUI, users can simply add the desired parameters to the YAML file (ex: studio/app/optinist/wrappers/caiman/params/caiman_cnmf.yaml) as needed.

9 ● I noticed you have a “use_cuda” option for caiman NoRMCorre. I would suggest

removing it to avoid user confusion since caiman’s GPU accelerated NoRMCorre was

written using skcuda which is no longer maintained.

We will remove the use_cuda option from the default parameter list and add the note in the documentation in the next update.

10 Use of optinist

Docker installation was seamless following these instructions:

https://optinist.readthedocs.io/en/latest/installation/docker.html

I have Docker version 24.0.2 which was released in May 2023, so relatively recent.

I tried to use it, I gave it the caiman demo movie and tried to do motion correction, this was the

workflow:

The caiman demo movie I’m referring to:

https://github.com/flatironinstitute/CaImAn/blob/main/example_movies/demoMovie.tif

I clicked “Run all” to run the workflow, but unfortunately I could not get further. I first thought it

hanged for some reason because even after 10 minutes it didn’t finish. This movie usually

finishes within seconds on my computer. ~15 minutes later it actually didn’t finish and this is the

traceback I got:

Thank you very much for trying it out. We are aware that errors are more likely to occur during environment setup, particularly with CaImAn. The latest minor update (v1.3.0) fixes the dependency issues in CaImAn. In addition, we have created a Docker all-in-one version (https://hub.docker.com/r/oistncu/optinist-allinone) with pre-installed environments for both CaImAn and Suite2P. This version should help avoid errors during environment setup.

11 I have important suggestions to improve this experience:

1. Have a way to install the components, such as caiman, before running the workflow so

that the user knows if the install will work. I thought that after pulling the docker image I

was ready to go.

We are going to separate construction of the environment and running pipeline in the next major version of OptiNiSt.

2. Format the traceback display and the output to error.log so that they are human

readable. Everything is just output as one line which makes it very difficult to read.

To make error reasons clearer, we will improve the readability of error logs and save them in an accessible format. Additionally, we plan to include troubleshooting guides and FAQs in the documentation in the next major update.

3. I’ve attached the full error.log with this review.

4. Have a way for users to easily monitor the output while a workflow is running.

In the new version, we make the backend terminal log to be posted to the GUI.

About the error itself, we can see that conda failed to create an environment. This is probably

due to the pinned dependencies mentioned earlier, and being pinned to python3.8. I didn’t go

through it fully but there seem to be a lot of dependency conflicts.

Unfortunately I couldn’t not try the software further due to these issues. If the developers can

address these issues I am happy to make another attempt to properly evaluate optimist from a

user’s perspective.

With that said, I do still support publication of this manuscript after these issues have been

addressed. Maintenance of scientific software is hard, and it is especially difficult in the calcium

imaging community.

Thank you for your positive feedback regarding our publication. We are also well aware of the challenges of maintaining a software like this in the calcium imaging field. Please try the Docker all-in-one version, which at least environment-dependent error should not occur for CaImAn.

Other comments:

I couldn’t find any way to set the number of processes to use when running a workflow, this is

quite essential.

The snakemake settings for controlling pipeline execution can be found in the icon at the top-right corner of the workflow interface, where you can specify the number of cores to use (figure bellow).

I wouldn’t put more effort into making it possible for users to add their custom modules. From

our experience users who are comfortable with programming are more likely to just write their

own scripts or notebooks than trying to do that within the constraints of any existing toolkit. This

also ties into creating tools with well-defined scope rather than a tool that tries to do everyting.

Even though adding a custom module requires familiarity with python coding, once a new module is created, that can be utilized by other users in the team or elsewhere. We also aim to encourage developers of analysis algorithms to provide their method as modules to be included in OptiNiSt.

**Response to the reviewer #2:**

OptiNiSt by Yamane et al is a new framework for creating calcium imaging data analysis pipelines.
I think OptiNiSt is a rather useful addition to the repertoire of platforms/frameworks that exist out there to help neuroscientists (and others) carry out their calcium imaging analysis. I largely agree with the authors that OptiNiSt will help neuroscientists to standardize

OptiNiSt 'packages' some of the standard tools (for cell detection, dimensionality reduction, etc) for calcium imaging analysis and appears to be expandable.
The use of YAML is a good choice and maybe offering a slight advantage over other platforms/frameworks that use JSON files or other options. The fact that OptiNiSt can store analysis results in NWB format is also another potential benefit for its users.

Thank you for your positive comments. In response to your feedback, we have revised the manuscript and updated the figures accordingly.

Minor comments:
1. Table 1: I think the paper would greatly benefit by having a figure where the various modules are listed in a nice visual way in catergories (e.g. cell detection, input, dimensionality reduction) instead of this long list in Table 1. Nobody is saying not to keep table 1 as a supplemental file but it is rather difficult to get a visual overview of the modules in the current way that they are presented.

We reformatted the information in Table as a Supporting information, S1 Table.

2. Figure 3 I think this figure needs some rearrangement to make the details of Panel A more visible and I think subheadings in panel B and panel C are required to explain to the reader what they are looking at. An alternative would be to move Panel A to supplemental and increase the sizes of panels B and C.

Fig3A was moved to the Supporting information S2_movie. The rest of the panels were reformatted with sub headings. Text explanations were also added to the main text (Line 365-)

3. Related to Figure 6 and Figure 7. It is not obvious to the reader how and to what extent a user can modify the parameters within individual modules (e.g. different motion algorithms) before running them and after (to optimize the motion correction/cell detection). A discussion of this would be important (I believe there is a single sentence on this on line 309).

Explanations of the parameter change (line 429- for Figure 6, 443- for Figure7) were added. We also clarified possible scenario to optimize the parameters (line 434-). In addition, we created some videos to show how to create pipeline, change parameters, and run (Supporting information S4 movie).

4. Figure 8: It is not clear to me whether the plots are interactive. E.g. can you click on the GUI on Figure 8 panel C data and go to the actual projection to visualize the quality of the segmentation and motion correction for example?

Unfortunatelly, in current setting, the interactive plots are only available for pre-installed modules for cell extraction (suite2P_cell_detection, caiman_cNMF and LCCD_celldetection). This point was clarified in the text (limitation section: line 520-).

5. I suggest that the authors also discuss at the end of the manuscript the limitations of the OptiNiSt.

We added the ‘Limitations’ section in the manuscript.

**Other changes:**

To meet the journal requirements, we slightly modified the composition of Figures 2, 3, and 4 and added a video clip as supplementary information.
