# Supplementary material for: Optical Neuroimage Studio (OptiNiSt): Intuitive, scalable, extendable framework for optical neuroimage data analysis
Source: PLoS Comput Biol. 2025 May 19;21(5):e1013087. doi: 10.1371/journal.pcbi.1013087 (PMC12124740; doi:10.1371/journal.pcbi.1013087)
Supplement: Attachment — Submitted filename: rebuttal_r2.docx [file pcbi.1013087.s010.docx]

Thank you for your advice on improving the software. The issues you have pointed out so far have been implemented as much as possible in the March update. We are currently checking for any issues related to the update, and we expect to complete this process by early April. Additionally, we plan to continue making improvements, including the remaining suggestions that have not yet been implemented, in future updates.

***1****. “Regarding data visualization and plotting, we have implemented lazy loading. For example, when visualizing a movie for checking purposes, only the first 10 frames are loaded by default. Similarly, for displaying extracted time courses, only the fixed number of time points are shown by default. Both cases are designed to allow reloading of specific portions of the data as needed.”*

Unfortunately this does not appear to be true. Lazy loading commonly refers to low latency random-access loading of segments of a file without loading the entire file into RAM; these files can be many times bigger than the RAM available on the system. Common methods for lazy loading in python are numpy.memmap (which caiman uses extensively for the algorithms, but can also be used to lazy load files for visualization), the dask library (which is used by minian, a 1-photon calcium imaging tool), zarr, and there are a few others.

I tried running motion correction on a 15GB movie with ~32GB of available RAM to test the lazy loading.

- When running optimist locally (i.e. server and client are the same machine) it copies the file and this takes time. It would be useful to note this for users, that the file is being copied under the hood.

- I set the number of cores to 8 in the Snakemake settings but it seems like this setting wasn’t followed, it still used all the cores and ran out of RAM on my machine. A 15GB movie can be motion corrected even if the system doesn’t have much RAM if fewer cores are used since fewer chunks of the movie will be motion corrected in parallel.

To check another way whether it supports lazy loading I tried to just visualize an existing memmapable tif file which can be opened lazily with tifffile.memmap https://github.com/cgohlke/tifffile/blob/f1d053c1f9922a207b84df615eefa52c6fbae3bb/tifffile/tifffile.py#L1461-L1482

Optinist actually loads the entire movie into RAM. I also searched through the codebase and couldn’t find anything that indicates lazy loading is used for input movies, nor whether caiman’s memmap motion correction movie is lazy loaded for visualizing. If i understand correctly, optinist uses imageio.volread for input movies which loads everything into RAM by default: https://imageio.readthedocs.io/en/v2.15.0/_autosummary/imageio.volread.html

My suggestion:Anyways, I would note in the docs that a limitation is that you cannot lazy load movies, which is fine for small movies that easily fit in RAM. Unless I am mistaken and you can lead me to the part of the codebase that performs lazy loading. Also it would be difficult to understand the CNMF results without being able to explore the movie across time while interactively looking through ROIs with a movie that has hundreds of components, but this is fine for smaller movies without a large number of components.

As the reviewer pointed out, OptiNiSt does not have a built-in feature to avoid loading all data into RAM using methods such as memmap. We added the issue in the limitation part in the manuscript (line 357-). We will explore ways to improve the handling of large-scale data in future development.

Regarding the number of cores setting in Snakemake, we have confirmed that it is effective for functions other than those in CaimAn. However, since CaimAn functions have their own parameters for specifying the number of cores, our implementation did not account for this, which likely caused the reported issue. We will address this in a future update.

*3. “While designs, specifications and testing of OptiNiSt are done by neuroscience researchers, actual coding is done by professional software development team that can create maintainable codes robust against personnel turnover, which actually happened in the course of development. We promote ease of maintenance by recording the development history.”*

Looked through the codebase (specifically through studio/app/common/dataclass, I did not go through the entire codebase in detail) and I noticed that there are no docstrings. Adding docstrings would help new developers understand the codebase quickly.

Currently, there were internal documents shared among engineers regarding the code structure, but they have not been made publicly available. We added the description of whole architecture in the documentation (https://optinist.readthedocs.io/en/latest/specifications/architecture.html). As you pointed out, we will consider improving the codebase by adding docstrings to make the overall structure easier to understand for developers.

Other comments:

Bug I noticed: After changing the image that I selected to load I had to delete the existing “caiman_mc” node and create a new one, because it just processed the prior video otherwise. I got a similar error with the cnmf node, I had to delete the node and make a new one if I ran it and then changed the parameters.

We have investigated the reported bug but were unable to reproduce it. In O**ptiNiSt**, the process is divided into two separate actions: **(1) uploading data** and **(2) selecting the data for computation**, each with its own button. Could it be possible that the data was not updated using the selection button (2) after uploading?

Issue template: an additional template in English would also be useful.

We have added an English version to the issue template.
